# A prospective randomized controlled study on the role of restoring liver diaphragm surface tension and pain control at port sites in optimizing pain management following laparoscopic cholecystectomy

**DOI:** 10.1002/ags3.12602

**Published:** 2022-07-26

**Authors:** Mohamed Lotfy Ali Abuelzein, Mohamed Ali Baghdadi, Waleed Ahmed Abbdelhady, Mostafa Mohamed Khairy

**Affiliations:** ^1^ Department of General Surgery, Faculty of Medicine Zagazig University Zagazig Egypt; ^2^ King Khalid Hospital Ministry of Health Riyadh Saudi Arabia

**Keywords:** gall stones, laparoscopy, pain, surface tension

## Abstract

**Introduction:**

After laparoscopic cholecystectomy (LC), pain is still a significant concern leading to extended hospital stays or readmissions. A standardized strategy is needed to offer effective pain relief postoperatively. The pain in the early postoperative period is mainly due to elimination of intraperitoneal surface tension. The aim of this study is to evaluate the restoration of intraabdominal surface tension and the use of bupivacaine‐soaked tachosil to control parietal abdominal pain at the port sites to optimize postoperative pain management.

**Patients and methods:**

Between March 2020 to December 2021, 816 patients undergoing LC were randomized into two groups after exclusion of 12 patients: Group A—interventional contained 402 patients; Group B—control contained 402 patients. Data to be compared were made in terms of operative time, shoulder pain, upper abdominal pain, and number of analgesic doses and hospital stay. Pain intensity was assessed by using the visual analog scale.

**Results:**

There was no significant variation in the demographic data between the two groups. There was significant statistical difference between Groups (A) and (B) regarding severity of shoulder pain and port site pain and number of analgesic doses and hospital stay in favor of Group (A). The results were evaluated within 95% confidence intervals and significance was determined as *P* < .05.

**Conclusion:**

The restoration of intraabdominal surface tension by absorbing as much CO_2_ as possible at the end of laparoscopic cholecystectomy via the epigastric port route, as well as the use of bupivacaine‐soaked tachosil to control parietal abdominal pain at the port sites; both steps significantly improved postoperative pain management, reduced the number of analgesic doses, and decreased the length of hospital stay.

## Graphical abstract

The cornerstone of treatment for symptomatic gallbladder stones is laparoscopic cholecystectomy. Pain is the commonest cause of extended hospital stay. Disturbance of liver—diaphragm surface tension is the main cause of pain. Retained CO2 is a major concern post laparoscopic cholecystectomy. Port sites pain is another contributor of the post laparoscopic cholecystectomy pain

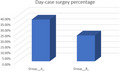

## 1 INTRODUCTION

The cornerstone of treatment for symptomatic gallbladder stones is laparoscopic cholecystectomy. After a laparoscopic cholecystectomy, pain is still a significant concern, leading to extended hospital stays or readmissions. Because analgesic procedures vary widely, a standardized strategy is needed to offer effective pain relief.^1^ Although laparoscopic cholecystectomy provides a number of advantages over open surgery, pain in the postoperative period remains a great concern. Postoperative pain, which requires injectable pain medications, prolongs the patient's stay in the hospital and is one of the obstacles to conducting laparoscopic cholecystectomy as a day case. The discomfort peaks within 6 hours after the operation and then progressively subsides over a few days, although it varies significantly among patients.^2^, ^3^ In 58%‐70% of patients, postoperative pain medications are required.^4^, ^5^

The pain that patients experience following laparoscopic cholecystectomy may be classified into three categories based on its origin: parietal, visceral, and shoulder pain.^6^

The parietal or somatic type of pain is generated by the abdomen wall being traumatized upon trocar insertion. The visceral type of pain is a result of the surgical dissection and manipulation of the gall bladder bed. Shoulder tip pain has been observed often, ranging from 35% to 63%, but the severity is significantly less severe following laparoscopic cholecystectomy than other types of laparoscopic surgery.^7^ While the visceral and parietal pain subside after 24–48 hours, shoulder pain may worsen.^8^ According to several studies, the majority of pain in the early postoperative period is of the visceral type. According to some, the primary source of overall abdominal discomfort is the incision sites, followed by the pneumoperitoneum, and finally the cholecystectomy.^9^

Numerous techniques have been attempted to alleviate postoperative pain, including gasless procedures, low‐pressure pneumoperitoneum, saline washout, subdiaphragmatic instillation of a local anesthetic agent, and local anesthetic infiltration at the port site wounds.^10^, ^11^, ^12^ This is in contrast to studies that demonstrate that infiltrating a local anesthetic drug into the trocar insertion site does not alleviate pain, suggesting that parietal pain does not contribute much to overall pain.^13^, ^14^

The cause of the shoulder pain is unknown; proposed causes include neuropraxia of the phrenic nerve, stretching of the subdiaphragmatic fibers due to the pneumoperitoneum, increased stretch on the liver's diaphragmatic attachments attributable to a decrease in visceral surface tension, and peritoneal injury as a result of chemical, ischemia, or traumatic injury.^15^, ^16^ In this study, we conducted active management steps on the case group in a trial to markedly decrease the post‐laparoscopic cholecystectomy pain in this group and compared the findings with those of the control group.

## 2 PATIENTS AND PROCEDURES

### 2.1 Guidelines

This study has been reported in line with Consolidated Standards of Reporting Trials (CONSORT) Guidelines.

### 2.2 Study place

This study was carried out in two big centers (general surgery department in Zagazig University Hospital, Egypt and King Khalid Hospital MOH, Saudi Arabia).

### 2.3 Study period

March 2020 to December 2021.

### 2.4 Source of data

Patients admitted with clinical diagnosis of symptomatic gall bladder stones.

### 2.5 Sample size

A total of 816 patients with a clinical diagnosis of symptomatic gall bladder stones were included in this study. Patients were randomly assigned to one of two groups: Group (A)—includes 408 patients as the intervention group (the group to whom our technique was applied); Group (B)—includes 408 patients as the control group.

### 2.6 Method of sampling

Simple random sample with a balance.

### 2.7 Method of sample size calculation

Taking the level of significance to be 5% and the power of the study to be acceptable at 80% with a confidence level of 95%, the sample size calculation was based on pain scores in similar studies in which patients were subjected to interferences to decrease the port site pain and reduce the residual CO_2_ volume.^17^, ^18^ Therefore, a total of 408 patients were included in each group.

### 2.8 Method of randomization

Patients were randomly allocated using a random sequence generator. Random allocations were sequentially numbered in sealed opaque envelopes, which were opened before surgery. Patients, clinicians, and researchers following the patients postoperatively and collecting data were blinded to the assigned group. Process of randomization was carried out by the registration office.

### 2.9 Inclusion criteria

Patients who were having symptoms consistent with biliary colic, had ultrasound evidence of gall stones, and were classified as American Society of Anesthesiology (ASA) I and II and with ages ranging from 18 to 65 years.

### 2.10 Exclusion criteria

Patients who refused to give consent, were pregnant, had a history of drug abuse, had CBD stones, acute cholecystitis, acute pancreatitis, previous abdominal surgery, a history of peritonitis, or had carcinoma of the gall bladder.

### 2.11 Procedures

After obtaining approval for the procedure, all patients in this research gave consent and operated on by the two experienced surgical teams, one in each center, and were anesthetized by two anesthesiologist teams who adhered to a predetermined procedure, one in each center. Vital signs were monitored in accordance with clinical norms. The postoperative assessment was performed by two independent clinician teams, one in each center.

All cases were performed via four ports. One 5‐mm port was placed via the umbilicus (camera), one 10‐mm port in the epigastrium at the level of the lower border of the right liver lobe and two 5‐mm ports were placed one in the right hypochondrial region in the midclavicular line 2 cm below the costal margin and one in the anterior axillary line at the level of the umbilicus. CO_2_ was the gas used for abdominal insufflation, using low flow rate of 2 L/min intraabdominal pressure was adjusted to 12 mmHg in both groups. After the formal triangle of Calot's dissection was performed and the cystic duct and artery were controlled, the gallbladder was removed via the epigastric port. The gallbladder bed is thoroughly inspected for bleeding or biliary leakage then:

- **In the control group (B),** all ports were removed under vision, cessation of insufflation was carried out, the umbilical port valve was opened, and the abdomen was gently squeezed to drain the retained CO_2_. The fascia of the epigastric port was closed with vicryl 0, and the skin of the four ports was closed with prolene 3/0.
- **In the interventional group (A),** the epigastric port was removed, and its site was inspected for bleeding, then reinserted and the laparoscopic suction device was introduced and left above the upper surface of the right liver lobe under the diaphragm. Then the midclavicular port and anterior axillary line port were removed under vision. Cessation of insufflation was done and the patient was kept in Trendelenburg position then the abdomen was gently squeezed to drain the retained CO_2_, the suction device was activated and sucked all the remaining CO_2_ from the celomic cavity till we met resistance (not liable to be blocked by omentum) leading the restoration of the liver‐diaphragm surface tension and the surface tension between the abdominal wall and viscera. Then the suction device was removed, and the epigastric port was blindly removed and the fascia of the epigastric port was closed with Vicryl® 0. Then four pieces of Tachosil® (one piece of 1 × 1 cm and three pieces of 0.5 × 0.5 cm) were impregnated with Bupivacaine® 0.5%w/v solution and applied in the subcutaneous region of each wound, and, lastly, the skin of the four ports was closed with Prolene® 3/0. Pain medications used postoperatively were pethidine 1 mg/kg intramuscular Q4HPRN and paracetamol 1 gm iv Q6HPRN in a unified sequenced alternative pattern. Statistical analysis was done using MedCalc® version 20.026.

Postoperative data to be compared were made in terms of operative time, shoulder pain, upper abdominal pain (incidence at 6,12,18 and 24 hours), number of analgesic doses, hospital stay, and number of days required to return to normal activity. Pain intensity was assessed by using the visual analog scale (VAS).^19^ The primary endpoint of this study is pain while, the total hospital stay, the total number of cases managed as day cases, and the total number of days required to return to normal daily activities are secondary endpoints. We used Post Anesthetic Discharge Scoring System (PADS) Score^20^ calculated by one member of our team who was blinded to the assigned group of each patient (Table 1).

**TABLE 1:** Post anesthetic discharge scoring system (PADS) score, scores of 9 or 10 mean the patient can be safely discharged

PADS score item	Answer choices	Pts
Vital signs	Within 20% of preoperative value	2
	20%‐40% of preoperative value	1
	>40% preoperative value	0
Activity and mental status	Oriented × 3 AND has a steady gait	2
	Oriented × 3 OR has a steady gait	1
	Neither	0
Pain, nausea and/or vomiting	Minimal	2
	Moderate, having required treatment	1
	Severe, requiring treatment	0
Surgical bleeding	Minimal	2
	Moderate	1
	Severe	0
Intake and output	Has had PO fluids AND voided	2
	Has bad PO fluids OR voided	1
	Neither	0

## 3 RESULTS

Between March 2020 and December 2021, 816 patients were evaluated for study eligibility. All were randomly assigned to either the intervention group (Group A) (n = 408) or the control group (Group B) (n = 408). Six patients were excluded from group (A) (Biliary leakage [n = 2], surgical site infection [n = 2], reactionary hemorrhage [n = 1], jaundice [n = 1]) and six patients were excluded from group (B) (atelectasis [n = 3], surgical site infection [n = 1], reactionary hemorrhage [n = 1], jaundice [n = 1]). Finally, 804 patients completed the study (113 males and 691 women). There was no significant variation in the demographic data between the two groups, regarding age, sex, and body mass index (BMI) distribution (*P*‐value >.05). The mean operative time in group (B) is slightly shorter, at (55.96 ± 9.43) minutes, than in group (A), which is (57.54 ± 9.56) minutes (*P*‐value .0186). The mean total hospital stay was statistically significantly lower in Group (A) at (1.2 ± 0.23) days than in Group (B) at (1.74 ± 0.54) days (*P*‐value .0001). Return to normal daily activity was statistically significantly lower in Group (A) at (5.27 ± 1.25) days than in Group (B) at (5.93 ± 1.43) (*P*‐value .0018) (Table 2).

**TABLE 2:** Demographic characteristics and clinical outcomes, a *P*‐value less than .05 (typically ≤0.05) is statistically significant

Groups	Group (A) n = 402	Group (B) n = 402	*P* value	Test used
Age [Mean (±SD)]	36.53 (±10.32)	38.26 (±12.34)	.0314	*t*‐ test
Gender				
Male number (%)	62 (15.4)	51 (12.68)	.621	Fisher's exact test
Female number (%)	340 (84.6)	351 (87.32)		
Body mass index (kg/m^2^) mean (±SD)	27.56 (±2.32)	27.43 (±2.17)	.4122	*t*‐ test
ASA classification				
ASA I number (%)	295 (73.4)	287 (71.4)	.5261	Fisher's exact test
ASA II number (%)	107 (26.6)	115 (28.6)		
Operative time in min mean (±SD)	57.54 (±9.56)	55.96 (±9.43)	.0186	*t*‐ test
Insufflated CO_2_ volume in Liters mean (±SD)	36.53 (±11.03)	35.74 (±10.42)	.2969	*t*‐ test
Total hospital stay (days) mean (±SD)	1.4 (±0.33)	1.74 (±0.54)	.0001	*t*‐ test
Day‐case surgery percentage	36.56%	22.63%	.0003	Exact Poisson test
Return to daily activities (days) mean (±SD)	5.53 (±1.33)	5.83 (±1.39)	.0018	*t*‐ test

**GRAPH 1:**
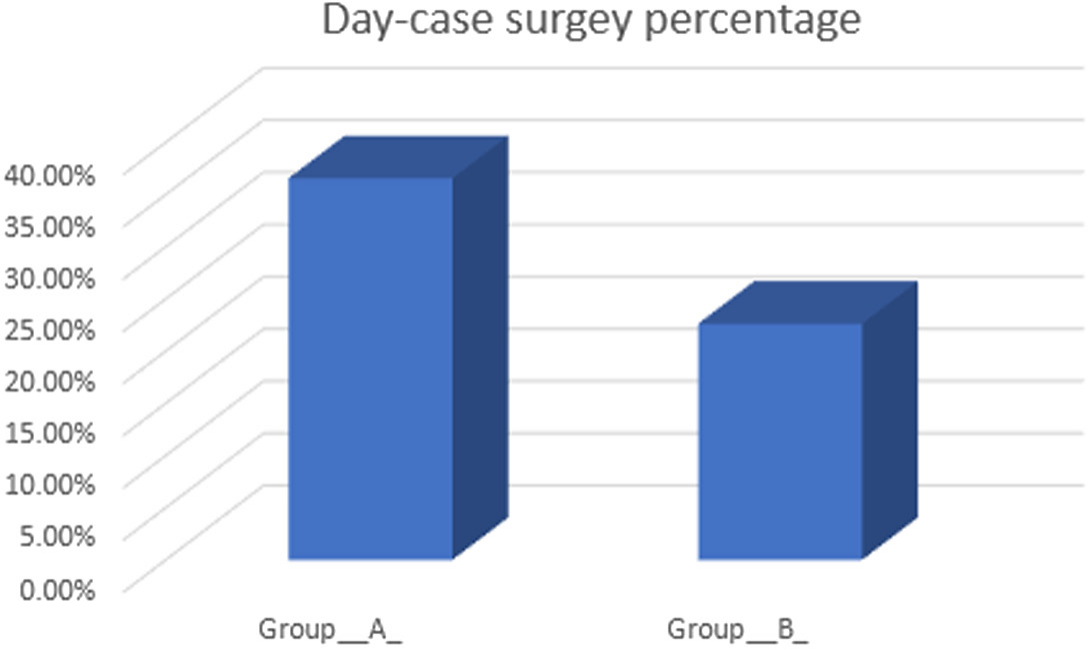
Day case surgery percentage in the two study groups

Due to the spread out of the data from the mean value we faced, we used the median value and the range to express and test the differences between the two groups. The median and the range of the registered score of VAS for patients in Group (A) at 6,12,18, and 24 hours were statistically significantly lower than those registered for group (B) for two of the assessed areas of pain perception (right shoulder and port sites) (*P*‐value ≤.05), While the median and the range of the registered score of VAS for the other two areas (left shoulder and interscapular areas) showed non‐significant difference between the two groups (Table 3). The mean number of doses of analgesics given in a specified period of time (0‐6 hours, 6‐12 hours, 12‐18 hours, and 18‐24 hours) postoperatively was statistically significantly lower in Group (A) than in Group (B) (Table 4). Due to the better pain control observed among the patients included in Group (A), a statistically significant number of cases were managed as day cases compared to those included in Group (B) (*P*‐value .0003) Table 2 and Graph 1.

**TABLE 3:** Types of pain of the two groups at each time point using VAS, a *P*‐value less than .05 (typically ≤0.05) is statistically significant

Groups	Group (A) n = 402	Group (B) n = 402	*P* value (Mood's median test)
Right shoulder pain VAS median (range)			
At 6 h	2 (0‐3)	3 (2‐4)	.0423
At 12 h	1.5 (0‐2)	2 (1‐3)	.0458
At 18 h	1 (0‐1)	2 (1‐3)	.0327
At 24 h	0.5 (0‐1)	1 (1‐2)	.0419
Left shoulder pain VAS means (SD)			
At 6 h	1.5 (0‐2)	2 (1‐3)	.0552
At 12 h	1.5 (0‐2)	2 (1‐3)	.0621
At 18 h	1 (0‐2)	1.5 (1‐2)	.0489
At 24 h	0.5 (0‐1)	1 (0‐1)	.0543
Port‐sites pain VAS means (SD)			
At 6 h	2.5 (1‐3)	4 (2‐5)	.0328
At 12 h	1.5 (1‐2)	2 (1‐3)	.0471
At 18 h	1.5 (0‐2)	2 (1‐3)	.0322
At 24 h	1.5 (0‐2)	2 (0‐2)	.0480
Interscapular VAS means (SD)			
At 6 h	1.5 (0‐2)	2 (1‐3)	.0236
At 12 h	0.5 (0‐1)	1 (1‐2)	.0512
At 18 h	0.5 (0‐1)	1 (0‐2)	.0672
At 24 h	0.5 (0‐1)	1 (0‐1)	.0437

**TABLE 4:** Number of analgesic doses, a *P*‐value less than .05 (typically ≤0.05) is statistically significant

Group	Group (A) n = 402	Group (B) n = 402	*P* value (Mood's median test)
Number of doses of analgesic [mean (range)]			
During the first 6 h	0.74 (0‐2)	0.92 (0‐2)	.0436
From 6‐12 h	0.48 (0‐1)	0.83 (0‐2)	.0342
From 12‐18 h	0.21 (0‐1)	0.62 (0‐2)	.0406
From 18‐24 h	0.11 (0‐1)	0.43 (0‐1)	.0212

## 4 DISCUSSION

The rising frequency of lengthy and difficult surgical procedures conducted ambulatorily creates new obstacles for immediate postoperative pain management. The capability to offer effective pain management utilizing easily available methods in the surgical day‐care patient setting may increase patient satisfaction and improve their impression of ambulatory anesthesia and surgery. Numerous techniques have been tried to alleviate postoperative pain, including systemic analgesics, regional blocks, local anesthetics, and preoperative dexamethasone injection (used to ameliorate nausea, vomiting, pain, and fatigue after elective LC).^21^ Despite the availability of effective painkillers, 30%‐70% of total patients experience significant postoperative pain. Intramuscular opioids administered on demand do not provide appropriate postoperative pain control in the vast majority of patients and significantly lengthen the recovery period. Pain after laparoscopy is the most commonly reported complaint, and the ability to alleviate it with easy and affordable methods would be useful.^22^, ^23^

The mechanism behind shoulder tip discomfort and diaphragmatic irritation is unknown and is believed to be complex. The duration of the pneumoperitoneum, the rate of gas insufflations, the peak intraperitoneal pressure, and the gas temperature have all been investigated. Distension, acidification of the intra‐abdominal environment, or temperature variations all tend to stimulate intraperitoneal nerve endings, which appear to be the common denominators. Insufflations may also trigger a localized inflammatory reaction that stimulates nerve endings and causes pain as a result of the original stimulation.^11^, ^24^, ^25^, ^26^, ^27^, ^28^ This may be reduced by lowering the rate of insufflations, decreasing the applied intra‐abdominal pressure, insufflating warmed and humidified carbon dioxide, and perhaps most importantly getting rid of as much gas as possible from the peritoneal cavity at the end of the operation.^6^, ^29^

Jackson et al investigated the relationship between post‐laparoscopy pain and pre‐discharge residual CO_2_. The study evaluated abdominal X‐rays to determine the amount of gas volume in the right subdiaphragmatic region. They found statistically significant relationships between the volume of retained gas and the intensity of the pain, indicating that leftover gas may be a substantial source of post‐laparoscopy pain.^30^

One study found that using a drain to remove leftover gas during the postoperative period reduced pain by 50%, but this result did not achieve statistical significance.^17^ In another study carried out by Koray Das et al, there was a significant difference in the intensity of the shoulder tip pain as compared to their control group. In this study, aspiration was performed with a flexible cannula that was inserted through the most lateral accessory port and positioned in the subdiaphragmatic space, but its holes can be easily blocked by omentum. It can also suck omentum, and hence it is liable to be trapped under the skin at the port site.^18^

However, in our study, the suction device was inserted via the epigastric port (the last port to be removed) and sucked all the remaining CO_2_ from the celomic cavity till we met resistance (it is not susceptible to be blocked by omentum), leading to the restoration of the liver‐diaphragm surface tension and the surface tension between the abdominal wall and viscera, just like the same principle of using the intercostal tube to treat pneumothorax and restoring the normally present surface tension between the visceral and parietal pleurae. We also got a statistically significant reduction in the median and the range of the pain score and a decrease in the mean number of analgesic doses in our interventional group (A) as compared to the control group (B). Also, there was a statistically significant reduction in the mean days of hospital stay decreasing the overall cost of the procedure and the mean days required to return to normal activities in our Group (A) as compared to our control Group (B). Also, the number of cases operated as day cases in Group (A) was statistically significantly higher than in Group (B), which had an additive positive economic impact. Our findings matched the results of Koray Das et al.^18^

In Tables 3 and 4, the spread out of the data from the mean value lead to standard deviations that became more than the means, so, we used the median value and the range to express and test the differences between the two groups.

Aman et al^17^ studied the effect of bupivacaine soaked Surgicel in the gallbladder bed to decrease post‐laparoscopic cholecystectomy pain. However, we used bupivacaine soaked tachosil to control parietal abdominal pain in the ports sites to extend the effect of bupivacaine and anesthetize the skin and deep parietal nerves. We got a statistically significant reduction in the mean pain score at the port sites and a decrease in the mean number of analgesic doses in our interventional Group (A) as compared to the control Group (B). TachoSil® is a collagen sponge coated with the human coagulation factors fibrinogen and thrombin. It is used during surgery to stop local bleeding in internal organs (hemostasis).^31^ Bupivacaine® typically begins working within 15 minutes and lasts for 2 to 8 hours,^32^ but tachosil impregnated with bupivacaine further extended the latter's duration and achieved a good pain control by anesthetizing the sensory nerves in the subcutaneous tissue, the sheath, the muscles, and the parietal peritoneum at the port sites in the first postoperative day. We just needed a piece that was 2 × 2 cm so we were using the small pack which contained two sterile 2.5 × 3 cm pieces (nearly costs as one single‐use port), which was sufficient for two patients.

In conclusion, the restoration of intraabdominal surface tension was carried out by absorbing as much CO_2_ as possible at the end of laparoscopic cholecystectomy via the epigastric port route, as well as the use of bupivacaine‐soaked tachosil to control parietal abdominal pain at the port sites. These two final steps improved postoperative pain management, reduced the number of analgesic doses, decreased the length of hospital stay and the overall cost. We strongly recommend ending every case of laparoscopic cholecystectomy with these two final steps.

### 4.1 Limitation

Further studies with larger sample size are recommended to support our findings and other studies are recommended to study the exact causes of postoperative shoulder pain in patients undergoing laparoscopic surgery in general.

## DISCLOSURE

Funding: This research did not receive any specific grant from funding agencies in the public, commercial, or not‐for‐profit sectors.

Conflict of Interest: The authors declare no conflicts of interest for this article.

Approval of the Research Protocol: The protocol for this research project has been approved by a suitably constituted Ethics Committee of our university hospital (ZU‐IRB #6534/15‐2‐2020).

Informed Consent: All informed consent was obtained from the subject(s) and/or guardian(s).

Registration at clinicaltrials.gov protocol registration quality control review criteria NCT05214157 https://clinicaltrials.gov/ct2/show/NCT05214157.
